# The Potential of Game-Based Digital Biomarkers for Modeling Mental Health

**DOI:** 10.2196/13485

**Published:** 2019-04-23

**Authors:** Regan Lee Mandryk, Max Valentin Birk

**Affiliations:** 1 Department of Computer Science University of Saskatchewan Saskatoon, SK Canada; 2 Department of Industrial Design Eindhoven University of Technology Eindhoven Netherlands

**Keywords:** digital games, digital phenotyping, mental health, computational modeling, big data, video games, biomarkers

## Abstract

**Background:**

Assessment for mental health is performed by experts using interview techniques, questionnaires, and test batteries and following standardized manuals; however, there would be myriad benefits if behavioral correlates could predict mental health and be used for population screening or prevalence estimations. A variety of digital sources of data (eg, online search data and social media posts) have been previously proposed as candidates for digital biomarkers in the context of mental health. Playing games on computers, gaming consoles, or mobile devices (ie, digital gaming) has become a leading leisure activity of choice and yields rich data from a variety of sources.

**Objective:**

In this paper, we argue that game-based data from commercial off-the-shelf games have the potential to be used as a digital biomarker to assess and model mental health and health decline. Although there is great potential in games developed specifically for mental health assessment (eg, Sea Hero Quest), we focus on data gathered “in-the-wild” from playing commercial off-the-shelf games designed primarily for entertainment.

**Methods:**

We argue that the activity traces left behind by natural interactions with digital games can be modeled using computational approaches for big data. To support our argument, we present an investigation of existing data sources, a categorization of observable traits from game data, and examples of potentially useful game-based digital biomarkers derived from activity traces.

**Results:**

Our investigation reveals different types of data that are generated from play and the sources from which these data can be accessed. Based on these insights, we describe five categories of digital biomarkers that can be derived from game-based data, including behavior, cognitive performance, motor performance, social behavior, and affect. For each type of biomarker, we describe the data type, the game-based sources from which it can be derived, its importance for mental health modeling, and any existing statistical associations with mental health that have been demonstrated in prior work. We end with a discussion on the limitations and potential of data from commercial off-the-shelf games for use as a digital biomarker of mental health.

**Conclusions:**

When people play commercial digital games, they produce significant volumes of high-resolution data that are not only related to play frequency, but also include performance data reflecting low-level cognitive and motor processing; text-based data that are indicative of the affective state; social data that reveal networks of relationships; content choice data that imply preferred genres; and contextual data that divulge where, when, and with whom the players are playing. These data provide a source for digital biomarkers that may indicate mental health. Produced by engaged human behavior, game data have the potential to be leveraged for population screening or prevalence estimations, leading to at-scale, nonintrusive assessment of mental health.

## Introduction

Playing games on computers, gaming consoles, or mobile devices (ie, playing digital games) has become a leading leisure activity of choice, with consumer spending on digital games exceeding US $134 billion [1] and outranking spending on music and movie tickets combined [2-4]. There are approximately 200 million gamers in North America, 354 million in Europe, 330 million in the Middle East and Africa, 234 million in Latin America, and 1.2 billion in Asia, which represents between 55% (Latin America) and 67% (North America) of the global online population [1].

When people play commercial digital games, they produce a lot of data—data that are not only related to play frequency, but also include performance data reflecting low-level cognitive and motor processing; text-based data that are indicative of affective state; social data that reveal networks of relationships; content choice data that imply preferred genres; and contextual data that divulge where, when, and with whom the player is playing. The game-based data produced by players are a rich source of information with the potential to be used for the assessment and modelling of health and health decline. In this paper, we argue that *game-based data from commercial off-the-shelf games can be used as a digital biomarker to assess and model mental and cognitive health and health decline*.

Assessment for mental health is performed by experts using interview techniques, adjacent to questionnaires and test batteries, and following standardized manuals [5]. However, there is interest in finding behavioral correlates that are predictive of mental and cognitive health and can be used for population screening or prevalence estimations [6]. When behavioral correlates are already known, researchers can develop *custom games* that are intended to place a player in a situation and monitor their response, response time, or performance. There are several examples of custom assessment games that have been developed to assess aspects of physical [7] and mental [8] health. For example, Sea Hero Quest [9], an internet game to track and assess dementia through navigational skills, can assess a large number of people very quickly, providing huge data sets with information on health decline over time for demographic groups and individuals.

Although there is great potential in custom games for assessment, we believe that the activity traces left behind by *natural interactions with digital games* can be used as a *digital biomarker* of health and health decline. As such, in this paper, we focus on the less-studied topic of data that can be gathered from “in-the-wild” gameplay of *commercial off-the-shelf games* and how natural gameplay data can yield insights into a person’s mental health.

Furthermore, although there are sources of data from games that may inform physical health or health decline (eg, identifying tremor development in patients with Parkinson disease from mouse kinematic data in a targeting-based game), we focus on the potential of game-based biomarkers in the context of *mental and cognitive health*. We consider the scope of mental health as defined by the Diagnostic and Statistical Manual of Mental Disorders, 5th Edition [5], which includes assessment criteria for mental disorders including developmental disorders (eg, autism spectrum disorder), neurodevelopmental disorders (eg, Parkinson disease), anxiety-related disorders, and depressive and personality disorders.

We first present a primer on games, the types of data, and the sources of data that are generated from natural digital gameplay. We then provide a description of five categories of digital biomarkers that can be derived from game-based data, including behavior, cognitive performance, motor performance, social behavior, and affect. We end with a discussion on the ethics, limitations, and potential of data from commercial off-the-shelf games for use as a digital biomarker of mental health.

## Primer on Games and Game Data

### Overview

Digital game play yields a variety of data that can be used to create novel digital biomarkers for health. Commercial games, as well as those created for research purposes, embed logging software that tracks interaction with the game. Game analytics—the tracking, analysis, and visualization of game-related data—is an important tool used by game designers, developers, and studios to inform about player experience [10-12]. Used both when games are under development as well as after release, game analytics leverage a variety of data types, the most common of which are player-generated data from interaction with the game [13].

**Figure 1:**
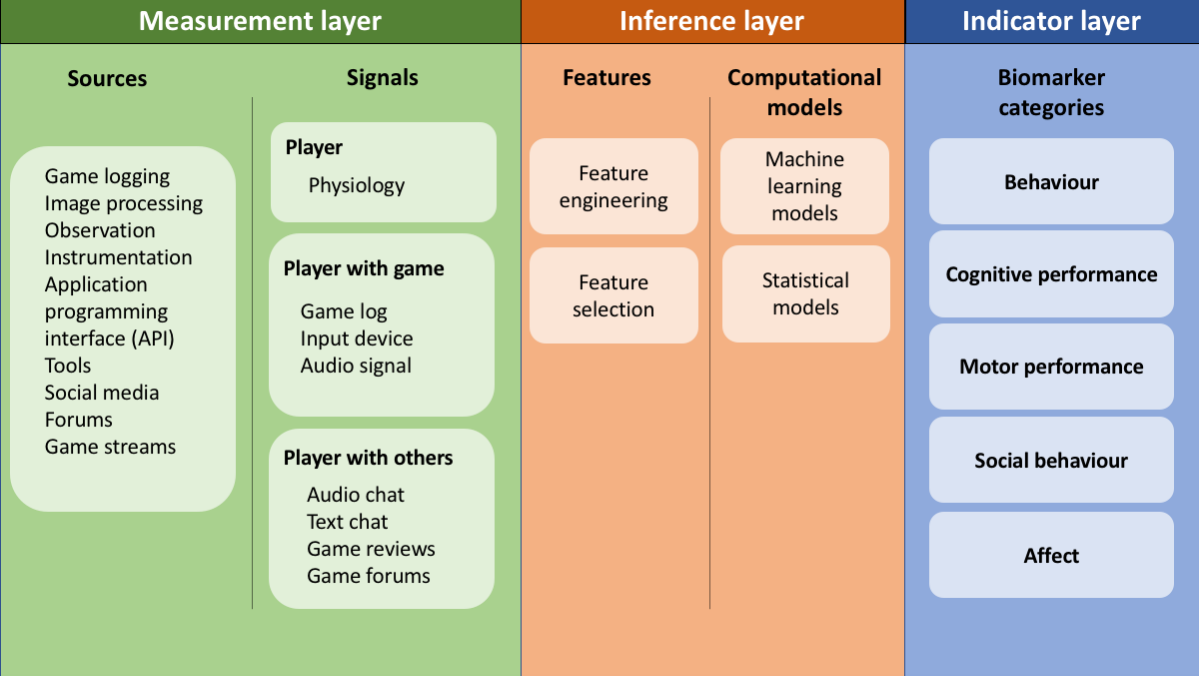
Machine learning pipeline for digital game-based biomarkers.

To understand how data gathered from gameplay can be used as a digital biomarker, we describe a standard machine learning pipeline [14]—starting with observations (eg, game log data), progressing to feature extraction based on the observed signals, and ending with predictions (ie, digital biomarkers)—in the context of gameplay. Figure 1 demonstrates this pipeline in the context of gameplay data and biomarkers for mental health. In the *measurement layer*, raw signals are gathered from various sources. In the *inference layer*, features are extracted and computational models are applied in an iterative process [14]. This computational modeling process results in final predictions, which are shown in the *indicator layer*. In this section, we briefly describe the measurement and inference layer; the next section focuses on the indicator layer and the five categories of game-based digital biomarkers that we propose.

### Measurement Layer

The first stage of generating digital biomarkers from game data is measurement of the data. Game data are available from a variety of sources, including the player, the player’s interaction with the game, and the player’s interactions with others within and around the game. Although the biggest source is derived from the player interacting with the game, data from the players themselves and from the players’ interactions with others can also provide value by building on research advances in digital biomarkers from nongame sources, for example, within the fields of affective computing [15] and natural language processing [16]. However, data from the interaction with the game is a novel source of signal with great potential for inference, but one that requires future research to interpret and understand.

#### Player Data

Player data are gathered from the players themselves. Signals such as heart rate, electrical activity of the brain, and galvanic skin response have all been explored in game contexts to infer player experience [17,18]. Although physiological data may not be easily accessible at the resolution gathered by medical-grade devices, novel methods of gathering physiological data in situ continue to emerge; sensors are embedded into game controllers for use in biofeedback games [19], to engage spectators [20], or simply as an innovative game input [21]. Further, physiological signals such as heart rate [22], blink rate, and facial expressions [23] can be derived from webcam-based signals along with facial expressions.

#### Player Interaction Data

Data from a player’s interaction with the game is primarily gathered from the game logs and includes high-level data (eg, number of active daily players) and low-level data (eg, each bullet fired in a shooting game, as was done in Gears of War 3 [Microsoft Studios, Redmond, WA; 2011]. Game developers insert logging software that tracks interaction with the game, and common metrics that derive from player interaction data include usage data (eg, login frequency, play time, and role choices), performance data (eg, win/loss data, death rate, kill rate, and score), and social data (eg, teammate records). Researchers can also use low-level input device data to infer information about the player. For example, the motion kinematics (eg, characteristics of the velocity or acceleration of a movement [24]) of the mouse or thumbstick movements could be used to infer information, or the pressure exerted on a screen in a touch-based game [25] or on the buttons of a game controller [26] could provide information on the experience state of the player. Finally, researchers could leverage the audio signals used in many online games to infer information; for example, environmental noise sensed through the microphone and coughing or respiration patterns of the player [27] could provide a rich source of data.

#### Data from Players’ Interactions with Others

Data from a player’s interaction with other players, spectators, or fans yield signals that are social in context, but can be used in the development of individual digital biomarkers. For example, text-based data from in-game chats could be analyzed using a keystroke dynamics approach, which leverages the timing and variability in typing rhythms (analyzing both time spent dwelling on keys and moving between keys, known as flight time) of common two-letter combinations (digraphs) and three-letter combinations (trigraphs) to infer the emotional state [28]. Further, sentiment analysis of social media posts for detecting suicidal ideation [29] could be modified and applied to text data generated in and around games. Text data are available from various sources, including in-game chat logs, game reviews, and texting about games on platforms such as Discord (Discord Inc, San Francisco, CA; 2015). Audio chat between players also generates a valuable signal; building on previous work that determines the emotional tone of the speech signal [30] might prove useful in the context of mental health modeling.

#### Gathering Game Data

Game data can be gathered from a single game; the majority of mobile games, as well as console games and computer games, record login and performance data as part of their game analytic engines. Much of these data are accessible to researchers through game application interfaces (APIs), including for the multiplayer online battle arena League of Legends (Riot Games, West Los Angeles, CA; 2009) [31], which boasts of 100 million active players monthly, or for the massively multiplayer role-playing games EVE online (CCP Games, Reykjavik, Iceland; 2003) [32] and Guild Wars 2 (ArenaNet, Bellevue, WA; 2012) [33]. However, in the context of modeling mental health, the aggregate play across game genres and titles may be more relevant.

Although aggregate usage data can be difficult for health researchers to gather (eg, PlayStation account data are not publicly accessible), there are methods by which overall play data can be gathered. Specifically, some game publishers provide an API to their suite of games; for example, battle.net features an API for a collection of games developed by Blizzard Entertainment [34] that includes the massively multiplayer role-playing game World of Warcraft, the role-playing game Diablo III, and the real-time strategy game Starcraft II, which is a popular title in the competitive electronic sports (e-sports) domain. Steam is the largest online portal for game play on computers (as opposed to dedicated gaming consoles), which features thousands of games and 100 million members; Steam has an API [35] that gives access to usage data and much more. Further, some data are available through other game portals such as Google Play [36] or Facebook Game Services [37], or through APIs that interface with games servers, such as the Sponge API [38] for the popular construction game Minecraft (Microsoft Studios, 2014). Further game streaming platforms (eg, twitch.tv) have recently been investigated as a potential source of data for research [39]. APIs generally provide limited access to the vast data available to the game publishers themselves, and collaborating with game companies to access richer datasets would be a valuable approach.

### Inference Layer

Computational approaches used to make predictions in data science derive inferences from features that are extracted from signals, which are observed in the previously described measurement layer. The quality of predictions derived from machine learning approaches (including, for example, deep learning [40] and clustering [41]) depends greatly on the quality of the features that are extracted from the signal. Selecting features and creating new ones [42] require expertise in the signals, their meaning, and the mathematical and computational approaches that are used in data science. Selecting and creating meaningful features (known as feature engineering [42]) are challenging and are the point in the pipeline where the expertise of the researcher makes a difference between a black box machine learning model (which blindly applies prediction algorithms to extracted data) and an informed expert-driven model that is built on theory, hypotheses, and iterative testing [42].

Although feature selection for some types of data is already well established in the literature (eg, sentiment analysis of text excerpts using natural language processing [16]), there are still open questions on how to best characterize signals derived from novel data sources such as video games. Game-based signal data require specific considerations, because players interact with the games on several levels (eg, explicit interaction with the game and implicit interaction around the game on third-party channels). A full description of the inference layer (or the machine learning approaches that are used within it) is beyond the scope of this paper; however, there are many standard resources to guide researchers who wish to leverage game-based signals to derive digital biomarkers of mental and cognitive health in the process of feature creation, feature selection, and machine learning [40,42-44].

## Game-Based Digital Biomarkers

### Indicator Layer

The data generated by digital games for player experience understanding can be used to extract features, from which researchers can predict characteristics of a player that may inform models of mental health. This prediction is part of the *Inference Layer* in Figure 1. In this section, we describe five categories of digital biomarkers for health that we feel can be extracted from game-based data—behavior, cognitive performance, motor performance, social behavior, and affect. In each section, we describe why this source of data is important for mental health modeling, the game-based sources from which it can be derived, and relevant literature that associates it with mental health.

### Behavioral Biomarkers

Behavioral biomarkers refer to metrics that are derived from the act of playing (eg, time spent playing and play frequency), high-level behaviors of players within a game (eg, role choices and play styles), and lower-level behaviors that reflect interaction with the game (eg, gaze fixation and low-level interface interactions).

#### Dosage as Behavior

In the context of health, frequency is often referred to as *dosage* and reflects both the overall engagement of the player with the game system and more complex patterns of engagement. For example, researchers may wish to know simply how much time is spent daily playing digital games (ie, quantity), but may also be interested in when gaming sessions occur, how long gaming sessions last, how much variability (ie, predictability) there is in the overall patterns of play—referred to as *entropy* in research on the computational analysis of human behavior [30]—or the context in which play is occurring (eg, where, with whom, and on which device).

Usage data, in terms of logins and play frequency, are available through in-game logging. There has been some research on the relationship between game play dosage and mental health from the perspective of addiction research. For example, research on pathological gaming has used definitions of addictive behavior to describe problematic or obsessive video game playing [45]; however, associations between excessive play and psychosocial health have not been consistently evident, even among gamers classified as “addicted” [46].

Although there may be meaning inferred from the overall dosage of play, there is likely even more richness in the patterns of play. For example, Lemola et al [47] showed that habitual computer game playing at night (between 10 pm and 6 am) is associated with increased depression scores, even after controlling for the total time played, suggesting that the timing of play could be predictive of mental health (eg, sleep-wake disorders). Further, research suggests that the context of play can moderate the associations of gaming behavior and well-being [48]. In-depth analysis of the contextualized patterns of play may indicate mental health; by considering variability in the timing, device, or location of play, there is likely predictive potential in behavioral data beyond dosage estimation.

#### Content and Preference Choices as Behavior

What we play and how we play tell us about who we are. Do we prefer games with complex, visually stunning, narrative heavy, open-world play (eg, Skyrim); games that gain their depth from complex interdependent choices (eg, SimCity); or games built around challenging puzzles (eg, The Witness)? When we play a specific genre, how do we play: Do we prefer to investigate every aspect of an environment or move through in-game quests quickly? When playing with others, do we play a supportive role (eg, healer) or do we prefer to play characters that drive progress (eg, damage dealer)? Do we play multiplayer games at all or do we prefer single-player games? Do we prefer to play games that are easy to progress through or do we choose ultra-hard modes to challenge our own abilities? There are several ways in which we can differentiate between different types of players [49], and the consideration of individual preferences that are observed through behavior may prove useful for identifying patterns related to mental health.

Research on predominantly male players who display problematic online play behavior (for example, play for long hours, aggressive behavior when forced to stop playing, and financial struggles as a result of play) shows that social anxiety is most prevalent among massively multiplayer role-playing game players and lowest for first-person shooter players [50], suggesting that genre preference could be an indicator of mental health. Further, self-discrepancy (ie, how well who we are aligns with who we want to be) research has shown that people with low mental well-being ascribe more desirable attributes (eg, kindness and creativeness) to a self-created character than to themselves [51]. Finally, content or preference choices that deviate from predictable patterns may indicate changes in life circumstances (eg, change in relationships or work status) that could contextualize other observed differences, which may help better predict and assess mental health.

#### Low-Level Game Interaction as Behavior

Play behavior is the composite of myriad low-level interactions, such as selecting a menu item or checking the health bar [52]. Low-level interactions that create events, such as firing a bullet, can be measured by including the logging code in the game software. Although low-level interactions such as clicking a button to fire a shot carry a signal, there is additional information in the interactions with the system itself (eg, moving the mouse) that precede the final event (ie, shooting). For example, we can learn about the visual cues a person responds to by analyzing eye gaze patterns or investigating mouse movement patterns during inventory search behavior. There is reason to believe that gaze patterns might help indicate mental health; for example, saccadic gaze behavior has been used extensively in the study of schizophrenia and bipolar affective disorder [53-55].

Measuring low-level interactions such as mouse movements or eye gaze requires either special equipment (ie, eye tracker) or needs to be implemented during development (ie, cursor logging software). Assessing low-level interactions during play of off-the-shelf games is challenging, because the game code cannot be modified to measure input (eg, click behavior), and bringing third-party equipment (eg, gaze tracking) to the user is logistically challenging and would influence play behavior. To overcome these challenges, researchers must determine ways to measure the signals unobtrusively. For example, research on older adults has leveraged image processing to investigate measuring cognitive performance through the card game Klondike Solitaire [8]. Although measuring low-level interactions is challenging outside of the laboratory context, there are many open questions about which player characteristics can be inferred. Does frequent non-goal–directed click behavior indicate nervousness? Can looking up the same in-game hint (eg, a password on a note in the inventory) multiple times indicate forgetfulness? Are quick changes in gaze fixation indicative of cognitive performance and information processing? The behavior that we show unconsciously might be hardest to access, but because implicit behaviors are difficult for players to influence, they have huge potential for inferring mental health.

### Cognitive Performance Biomarkers

Many games incorporate cognitive challenges such as memorizing sequences that rely on short-term memory (eg, Shadowrun), making decisions under certain conditions (eg, first-person shooter games), recognizing patterns (eg, Bejeweled), or analyzing complex information (eg, SimCity). Game difficulty can generally be adjusted (or adjusted dynamically), so that the cognitive performance required to succeed is well matched to the player’s abilities [56]. When players tackle cognitive challenges in games, they generate performance data such as the number of attempts, time spent to overcome a challenge, reaction times to stimuli, or the highest level they were able to complete without failure. There are a variety of game sources from which one can identify and measure cognitive performance in games. Leaderboards and achievements are aggregated indicators of game performance [57]: Leaderboards provide a high score in comparison with other players or personal bests, whereas achievements represent specific challenges a player has overcome, such as a challenging attack sequence, collecting a specific set of items, or taking out a certain number of enemies. Other performance indicators are ranks or titles of a player (eg, Platinum or Diamond rank in League of Legends), the rarity of owned items (eg, Stunted Direhorn in World of Warcraft), or a player’s player-vs-player statistic; however, these performance indicators may be more related to time investment than to the underlying cognitive ability.

To access cognitive performance biomarkers through games, we can look to specific game mechanics that map well to cognitive abilities. For example, the number of dodged hits in a fighting game is indicative of a player’s ability to anticipate and react, a difficult skill prevalent in elite athletes [58] and reduced in people with depression [59]. The turn-based role-playing game Shadowrun: Hong Kong (Harebrained Schemes, Seattle, WA; 2015) features a two-staged hacking challenge that requires players first to repeat an increasingly difficult sequence of numbers—a common task for assessing short-term memory [60]. The puzzle game The Witness (Johnathan Blow, 2016) presents challenges that require spatial rotation and abstract thinking (eg, one puzzle requires players to first identify the path through a three-dimensional maze and to then redraw the pathway on a two-dimensional input device).

Cognitive performance is an indicator of many mental health issues such as depression, anxiety, Alzheimer disease, Parkinson disease, or attention-deficit disorders [5]. People experiencing symptoms of depression, for example, show differences in executive functioning, sustained attention, and memory [61,62], which debilitates a player’s ability to play games that rely on these cognitive systems. Digital assessment software such as Cambridge Neuropsychological Test Automated Battery [63] have shown that many of the resulting divergences in cognitive performance related to mental health can be detected through simple tasks that are likely mirrored in digital games. Further, performance in games can be used to naturally assess cognitive abilities such as attentional [64] or visuospatial [39] biases.

### Motor Performance Biomarkers

Most games require motor input to interact with them. The input device used in games varies, but is generally comprised of touch input (ie, in mobile games); mouse and keystroke input (ie, in desktop games); and controller input, which consists of buttons to press and thumbsticks or mini joysticks to control (ie, in console games). Gaming consoles additionally sometimes have cameras that capture the user’s movements (eg, Microsoft Kinect and Sony PlayStation Camera). The level of motor interaction needed to play a game varies widely: Many games require complex sequences of input (eg, Street Fighter), whereas others take a very simple motor action, but require it to be completed quickly and repeatedly (eg, clicking in Cookie Clicker) or in combination with cognitive choices (eg, keystrokes in Starcraft).

Motor data in games can be gathered through the types of sources listed in the Measurement Layer section; however, researchers interested in millisecond accuracy of motor input can also write third-party logging software that captures interaction with the device [28]. Leveraging research in human-computer interaction, the kinematics of mouse movements (eg, velocity, acceleration, and percentage of time spent decelerating [24]) or the variability in mouse kinematics could be indicative of mental or cognitive health. Further, keystroke dynamics (ie, the low-level timing of typing actions) have been used to predict the stress of the typist [28,65], which could potentially inform stress-related mental health disorders. Game console cameras could be used to detect players’ movements, which has been associated with various emotional states [66] that might be indicative of mental health or health decline. In addition, the pressure exerted on gaming controls has been linked to frustration for both button presses [26] and touch input [25], which provides relevance in terms of players’ resilience to stressful stimuli. Leveraging an entire touch gesture can also provide interesting signals; for example, in Fruit Ninja gestures, timing and pressure features were used to discriminate low and high arousal and valence, both of which are relevant in the context of mental health [67].

Motor performance data have particular potential as a digital biomarker for neurodegenerative diseases such as Alzheimer, Parkinson, and Huntington diseases, which are characterized, in part, by psychomotor decline [5]; monitoring patients’ motor performance in games over time could reveal valuable information about the rate of disease progression.

### Social Biomarkers

Social rather than individual play is quickly becoming the dominant form of digital game play: Gamers spend an average of 6 hours/week playing with others online and 5 hours/week playing with others in person [68]. When people play digital games with others, they generate social behavioral data (for example, with whom are they playing and what role did they take in that interaction) as well as social communication data (for example, what words were exchanged and in which channels).

In terms of social behavior, some multiplayer game APIs (eg, League of Legends) provide information on who people were playing with; more subtle cues of social interaction, such as being part of a guild (eg, in World of Warcraft), the number of social contacts as compared to population averages, choices of predefined texts (eg, “good game”) sent (eg, Hearthstone), or the ratio of the type of games played (eg, single-player games vs team-based games) could be indicative of mental health. Communication-based game data can be drawn from primary sources such as in-game chats, forum posts, or voice-over-internet protocol applications (eg, Discord or Team Speak) and secondary channels such as social media (eg, Reddit, Twitter, and Facebook) or video commentary (eg, Twitch and YouTube). Natural language processing approaches allow researchers to identify word categories used and gives insights into the way we use language, for example, the ratio of self-references to social references in written texts [69]. Further, sentiment analysis allows us to gauge the valence of an attitude (ie, positive, neutral, or negative) toward a specific topic. Applied to written text or spoken words from game-based sources, these techniques provide insights into how users present themselves when interacting with others.

Social behavior is an important indicator in many mental health disorders and therefore important for health modelling. People suffering from depression, for example, show higher use of negatively valenced phrasing [70], a tendency to use more self-referencing words such as “I” or “me” [70,71], and use of fewer emoticons [72]. It also matters with whom they have contact; research on game play suggests that playing socially may be linked to well-being [73-75]. In game play, this might be reflected in the consistency with which we play with other specific players.

Although online communication has been found to negatively affect one’s well-being despite its social nature [76], the issue is more complex and requires nuanced consideration of the usage purpose, context, and individual differences [77,78]. A large proportion of research on digital biomarkers to predict mental well-being has focused on depression and anxiety; however, psychiatric research [6] suggests that the range of mental illnesses reflected in digital traces also applies to mental illnesses such as borderline disorder or bipolar disorder. Bipolar disorders might, for example, be reflected in increased parallel conversations, high levels of text output, and fluctuation of social relationships during up swings, while down swings would be characterized by disengagement and negatively valenced communication patterns.

### Affective and Emotional Biomarkers

Quantifying the emotional state of the player has been of interest to affective computing researchers and game designers who wish to understand or improve player experience [79]. Researchers detect affect using physiological signals [80], behavioral signals (eg, posture [81]), speech signals [30], eye gaze and fixation data [82], and sentiment analysis of text data [69].

Within game play, sources of measurement for affective biomarkers include in-game chat (text data), in-game audio for communication during online play, audio during streaming (eg, on twitch.tv), and the input data discussed in relation to motor performance biomarkers. In the game, there are also game-based text data that can be gathered from forums and chat platforms (eg, Discord) as well as game-related posts on social media. Physiological data are not necessarily straightforward to gather in the context of commercial off-the-shelf computer game play; however, recent advances in sensing over a distance, such as heart rate [83] or facial expression [84], gathered via webcam and increased prevalence of physiological input devices in commercial games [21] suggest that third-party logging could be used to link physiological sources with game APIs.

Understanding the emotional experience of players is of particular interest in the context of mental health, as many prevalent mental health issues (eg, depression and generalized anxiety) are closely tied to emotional wellness or can be predicted by aberrant responses to specific stimuli (eg, posttraumatic stress disorder) or in-game self-representations (eg, anorexia nervosa). For example, eye gaze patterns have been used to characterize individuals with various depression and anxiety disorders [85]. Impulse control and conduct disorders are characterized by difficulty in the self-regulation of emotions and behaviors [5], whereas emotional self-regulation difficulties are also characteristic in some developmental disorders (eg, autism spectrum disorder [5]).

## Discussion

### Overview

Digital biomarkers are increasingly being used to indicate potential mental health issues [86-89]. Computational phenotyping—the digital quantification of disease phenotypes—extracts the observable traits (eg, morphology, development, and behavior) of an entity from data sources that can be complex and heterogenous. In this paper, we propose using data generated from natural play of off-the-shelf digital games (ie, *digital biomarkers*) as one such complex data source. Five categories of game-based digital biomarkers—behavior, cognitive performance, motor performance, social behavior, and affect—were argued to include observable traits that could be indicative of mental health or health decline.

### Multiple Data Sources and Sensor Fusion

Each of the five described digital biomarkers mentioned has the potential to indicate mental health or illness; however, it may be in their combination that the true power of game data as a digital biomarker of mental health can be seen. Further, combining game data with other observable traits derived from smartphone data (eg, geolocation, accelerometer, and Bluetooth devices), sentiment from social media (eg, Google search, Reddit, Twitter, and Facebook), or physiology from integrated trackers (eg, Fitbit) may allow for rich and predictive models of mental health that leverage sensor fusion (ie, the use of multiple sources of data in combination [42]) for accurate modeling. We do not suggest that game-based data can be the sole biomarker for mental health in isolation, but that considering its inclusion in a suite of behavioral indicators may improve modeling in the context of mental and cognitive health.

To interpret data generated by different sources, data collected from a large sample with the intention to create a norm has several advantages. Sea Hero Quest [90], for example, generates insights into humans’ general capability to navigate and then identify behavior that deviates from norms as an indicator for early onset dementia. Establishing norms in game-based biomarkers is a complex undertaking but may have value when looking for deviations from norms as an indicator of mental health decline.

### Limitations

There are various factors that may compromise the predictive potential of game-based digital biomarkers. For example, pharmaceutical treatment or remission through therapy can interfere with accurate prediction: In terms of impulsivity, depressed people in remission are often grouped with control subjects rather than depressed participants [91], whereas in terms of visual acuity, depressed participants, regardless of the pharmaceutical treatment, are differentiated from the control group, but not each other [92]. As use of game-based biomarkers is a novel approach, researchers must carefully establish whether and how treatment or remission affects the behavior underlying the biomarker. Further, as with all digital biomarkers derived from in-the-wild data sets, events unrelated to the characteristic being predicted can greatly interfere; for example, a player who is on holiday, has a cold, has an upcoming deadline, or is experiencing harsh weather can exhibit behaviors that appear to be erratic, but which are driven by circumstance and not mental health.

Another limitation is that game-based biomarkers will only be relevant for assessment of populations who play games. People of all ages play games; however, preferences for various genres and platforms change with age [93]. A greater proportion of people of color in America play games, identify as gamers [94], own a gaming system, and form a faster-growing market than their white non-Hispanic counterparts [95]. In addition, almost half of all gamers self-identify as female; however, social gaming and mobile gaming are more important to female gamers than male gamers [96]. Demographic factors will need to be accounted for in any model of mental health built on game-based biomarkers. Finally, research on game-based biomarkers is in its early infancy: In this paper, we hypothesize that the potential biomarkers are based on existing scientific literature; however, significant research is needed to demonstrate the efficacy of game-based biomarkers and to identify specific game-based biomarkers for specific groups of mental health disorders.

### Ethics, Privacy, and Legal Use of Game Data

The use of data derived from digital sources is part of a larger discussion [97-99]; as with all digital data, game-based biomarkers require consideration for topics such as inferring identity, communicating mental health assessment, privacy of in-game conversations, and legality of gathering data unobtrusively.

Rare events in game-based data (eg, a difficult-to-unlock achievement) allows for the identification of individual players. As a result, researchers need to consider information reported on their players. When working with public players such as e-sports athletes or streamers, the in-game identity can be publicly linked to the actual identity of a player, which therefore requires considerations for privacy.

Technologies such as Mindstrong Health Services [100] or Facebook’s Suicide detection [101] show how data gathered from digital sources can be used to infer mental health and reach out to individuals who are at risk. Game-based biomarkers could result in similar services. However, communicating detected potential mental health issues might have negative consequences for users; for example, it is unclear what the consequences are of false positives (ie, a player being urged to seek help when there is actually no issue at all). Researchers need to consider the potential consequences of reporting mental health to players and the methods by which they communicate with players about the detected mental health issues.

Forums, in-game chats, and game streams require extra attention when analyzed, because users do not intend to have their information stored and may use phrasing and communication patterns that misrepresent conversations out of context. Consent to allow researchers to monitor conversations needs to be given explicitly, and player-initiated removal of unwanted data from further storage (eg, the use of a derogative term) requires consideration.

In addition to standard ethical considerations of data-driven inference, game-based data have characteristics that require special attention. First, the expectation that mental health could be inferred from a player’s pressure profile of a button press during game play is lower than that from explicitly posted texts about suicidal ideation on social media platforms [101,102]. Players should be clearly notified of the implications of implicit assessment from natural in-game behaviors that are, in the player’s view, unrelated to mental health. Further, extensive play for the purposes of assessment should not be a “slippery slope” into pathological play behaviors. Second, players often behave differently in games by enacting fantasies [103], trying out different personalities [104], or behaving in ways congruent with gameplay, which are incongruent with out-of-game expectations, such as acting violently or ultra competitively. Applying value judgements from models of behavior generated out of games may not apply within games and could increase the risks of false positives if not properly considered.

### Conclusions

Mental illness has become a major disease burden globally: Depression is currently the leading cause of disability worldwide [105]. Untreated mental illness has serious consequences; the estimated US $2.5-8.5 trillion globally in lost output attributed to mental, neurological, and substance use disorders is expected to almost double by 2030 [106]. In addition to these financial costs, people experience costs to their well-being that range from a lower quality of life [107] to a loss of life [108]. The emergence of smartphone and wearable devices has begun to show promise for the assessment of mental health, for example, for real-time assessment of suicidal thoughts [109] or acute phases of psychosis among people with schizophrenia [110]; however, there is additional work to be done before researchers can reliably use game-based biomarkers to predict a decline in a person’s mental health, such as the onset of a depressive episode, the progression of dementia, or behavioral changes that are related to social anxiety.

In this paper, we argued that owing to the prevalence of digital game play, there are several untapped sources of data, including behavior, cognitive performance, motor performance, social behavior, and affect. Further, we proposed that due to existing statistical associations between these five game-based digital biomarkers and mental health, there is untapped potential in game data for computational modeling that predicts mental health and mental health decline.

## Abbreviations

API: application programming interface
e-sport: electronic sport
